# Long-term cabergoline use does not predict degree of prolactinoma fibrosis nor significantly impact surgical outcomes

**DOI:** 10.1007/s11102-025-01628-8

**Published:** 2026-01-12

**Authors:** Adam N. Mamelak, Rachel Fox, Yaakov Rosenberg, Daniel Gomez, Yujie Cui, Anat Ben-Shlomo, Artak Labadzhyan, Ning-Ai Liu, Vivien Bonert, Odelia Cooper

**Affiliations:** 1 Department of Neurosurgery, Cedars-Sinai Medical Center, 127 S. San Vicente Blvd., Ste. A6313, Los Angeles, CA 90035 USA; 2 Pituitary Center, Department of Medicine, Cedars-Sinai Medical Center, Los Angeles, CA USA; 3 Biostatistics Shared Resource, Cedars-Sinai Medical Center, Los Angeles, CA USA

**Keywords:** Prolactinoma, Cabergoline, Dopamine agonist, Collagen volume fraction, Cavernous sinus invasion, Remission

## Abstract

**Purpose:**

Prolactinomas are often treated initially with dopamine agonists (DA). For patients subsequently treated with surgery, the effect of cabergoline (CAB) on tumor fibrosis and its potential impact on surgical outcomes is largely unexplored.

**Methods:**

Records of patients with prolactinoma treated by a single surgeon between 2006 and 2024 were examined. Analyses considered relationships among duration and cumulative dose of presurgical DA (DA + vs. DA-), extent of fibrosis measured quantitatively by collagen volume fraction (CVF) and qualitatively by surgeon assessment, and remission status at last follow-up.

**Results:**

Of 59 patients, 22 were DA- and 37 were DA+, including 29 treated only with CAB and 8 treated with CAB and bromocriptine. There were 44 macroadenomas, 13 microadenomas, and 2 giant adenomas; 28 had cavernous sinus invasion (Knosp grade 3–4) 52.5% were in remission at last follow-up. Median cumulative CAB dose was 79.3 mg (range, 5.4–6711), used for a median duration of 570 days (range, 16-7830). Neither CAB dose nor duration correlated with CVF (r^2^ < 0.01, p = NS). Both surgeon fibrosis assessment and CVF were higher in DA + patients, but neither independently predicted remission. Cumulative CAB dose and duration also did not predict remission. On univariable analysis, cavernous sinus invasion (OR 10.3, *p* < 0.001) and tumor size (OR 6.6, *p* = 0.02) predicted remission, but in multivariable analysis no single factor remained significant.

**Conclusion:**

Duration and cumulative dose of presurgical CAB use do not correlate with quantitative measures of tumor fibrosis and do not reliably predict the degree of fibrosis at surgery or the likelihood of surgical remission.

## Introduction

Prolactinoma is the most frequently encountered pituitary adenoma, representing approximately 50% of all tumors diagnosed [1, 2]. Prolactinomas are exquisitely sensitive to the effect of dopamine agonists (DA), and treatment leads to tumor shrinkage and normalization of prolactin levels in approximately 80–95% of patients [3, 4]. Because response rates are so high, medical management with DAs such as bromocriptine (BRC) or cabergoline (CAB) has generally been considered the preferred initial treatment strategy for most patients, with strong data to suggest that outcomes with CAB are superior to those with BRC [5, 6]. Intolerance to medication side effects, development of DA tumor resistance, poor patient compliance, and cost are all noted limitations to long-term DA use [1, 2, 7, 8] and may lead to a preference for surgery.

Surgical removal of the tumor has good long-term cure rates. For intrasellar microadenomas, reported cure rates are in the range of 70–95% [9, 10]. Even intrasellar macroadenomas that have not invaded the cavernous sinus (CS) have reasonably high cure rates of 40–60% [11]. Using modern endoscopic techniques that have low morbidity and mortality, surgical intervention can provide a rapid and immediate cure.

Long-term DA use, particularly BRC use, is thought to induce tumor fibrosis [7, 12]. This, in turn, may yield more difficult tumor removal when surgery is attempted, with resultant higher risk of damage to the pituitary gland or surrounding structures and reduced chance for long-term surgical remission. Several studies have assessed surgical outcomes in patients treated with DA, but most do not detail the duration and dose of DA used, quantify fibrosis, and/or provide follow-up clinical data and postsurgical remission status [13–15]. Where these results are available, data are almost exclusively limited to BRC use. A recent report investigating the relationship between BRC usage and tumor fibrosis in a large series of 290 patients concluded that BRC use increased tumor fibrosis in a dose-dependent and cumulative fashion, resulting in less early remission for microadenomas and more complications compared to patients with no tumor-induced fibrosis and/or less use of BRC [16]. However, the authors did not report longer term remission or recurrence rates.

CAB is far more frequently used than BRC in North America and Europe due to its better tolerability, requirement for less frequent dosing, and better rates of PRL normalization [5]. Yet, to date, fewer rigorous studies have evaluated the effect of CAB on tumor fibrosis almost and nearly all used qualitative assessment of tumor fibrosis by surgeons rather than quantitative analysis of histology [12, 17], rendering them prone to selection bias. One study of 4 patients quantified the extent of fibrosis in tumor samples and considered both dose and duration of CAB used [14], but surgical outcomes were not reported and there was no long term follow up.

Because both DA and surgery are effective as initial treatment modalities, especially for intrasellar microadenomas, debate has emerged regarding the most beneficial first-line strategy for prolactinomas [1]. We sought, for the first time, to quantitatively assess CAB dose, tumor fibrosis, and long-term surgical outcomes in a large series of patients with prolactinoma treated with or without presurgical CAB. We further sought to identify predictors of surgical remission and to correlate tumor fibrosis with clinical endpoints. Based on previous studies with BRC, we hypothesized that prolonged presurgical CAB use would induce measurable tumor fibrosis and adversely affect postsurgical remission.

## Materials and methods

### Study design

The surgical database of a single surgeon (ANM) was reviewed to identify all patients who underwent surgery for prolactinoma between January 2006 and April 2024 at Cedars-Sinai Medical Center, a quaternary referral center for pituitary disease. The electronic medical records of these patients were reviewed to identify demographic features, including sex, age at time of surgery, presenting symptoms, and surgical indications, as well as presurgical DA dose and duration of use, surgical findings, and surgical remission outcomes. Inclusion criteria included histological evidence of prolactin (PRL) immunostaining, documented serum PRL level > 100 ng/mL before treatment [18, 19], and at least 3 months of postsurgical follow-up [20–22]. Patients with adenomas co-secreting growth hormone were excluded. Tumor size (giant, macroadenoma, microadenoma) and presence of CS invasion according to the modified Knosp grading scale [23] were recorded based on pre-surgical MRI. Knosp grades 3–4 were considered invasive, while grades 0–2 were considered non-invasive. DA resistance was defined as an inability to normalize PRL and/or demonstration of increasing PRL levels or tumor size despite increasing doses of DA [24].

### Dose and duration of presurgical DA treatment

For patients who used DA prior to surgery, classified as DA+, DA type (CAB vs. CAB + BRC), duration of use, and total cumulative dose were recorded. Patients who only took BRC were excluded. No patient took CAB and BRC at the same time, and any patient that received BRC was later switched to CAB such that CAB was always the last medication taken and was always taken for the longer duration. If a patient took more than one DA, the dose for each was calculated. Patients who received no DA were classified as DA-. If DA was used for less than 2 weeks due to side effects or poor compliance, the patient was considered naïve to DA (i.e., DA-) barring contradictory data.

Cumulative DA dose was calculated using the formula: Cumulative DA dose in mg = (total number of days on DA per week) × (dose per day in mg). Patients who were treated with BRC and then switched to CAB were included in the DA + group. CAB only and CAB + BRC groups were separated out for some sub-analyses.

### Histological analysis

**Fig. 1:**
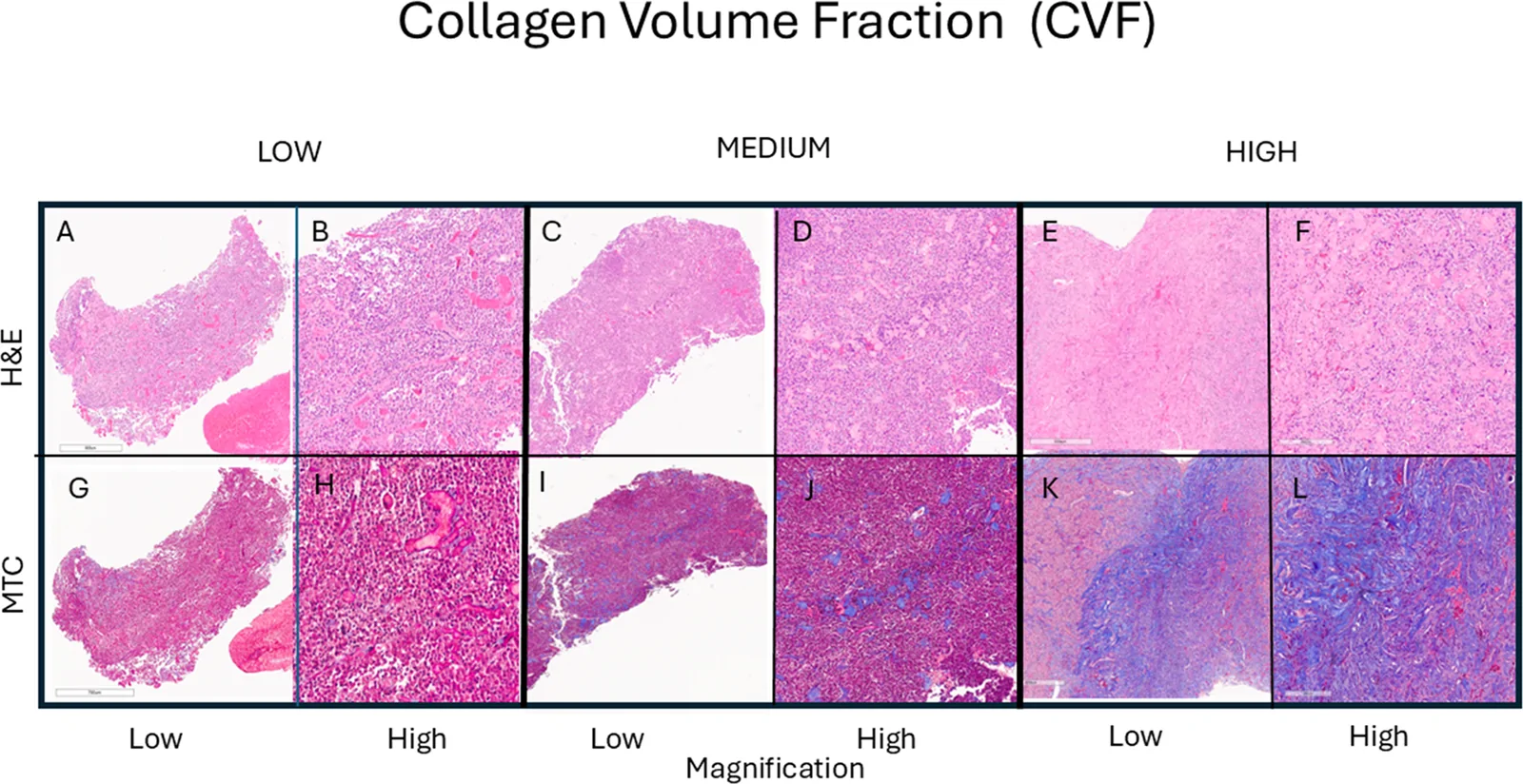
Representative examples of (**A**-**F**) histology (H&E) and (**G-L**) degree of fibrosis as measured using Masson’s trichrome stain for collagen. Both low and high power magnification is shown for each set (**A**,** B**,** G**,** H**), (**C**, **D**,** I**,** J**), (**E**, **F**, **K**, **L**). Increased percentage of blue staining on the MTC specimen indicates higher collagen content and, in turn, a greater degree of fibrosis. Tumor tissue shown with (**G**, **H**) low (CVF = 0.23), (**I**, **J**) intermediate (CVF = 16.7), and (**K**, **L**) high levels of fibrosis (CVF = 63.8)

Tissue blocks of paraffin embedded tissue from surgery were sectioned and stained with hematoxylin & eosin to confirm histology, and Masson’s trichrome (MTC) to assess degree of fibrosis (Fig. 1). Slides were converted to digital images and MTC-stained slides were subjected to quantitative analysis to measure degree of collagen staining (blue) relative to tumor (red) using ImageJ software (imagej.net). The process of calculating CVF staining was performed in a single batch analysis using a custom written macro in ImageJ (script available upon request).

Custom thresholding of red and blue intensity resulting from the MTC stain was applied for each image to account for differences in staining intensity.

Collagen volume fraction (CVF), or extent of fibrosis, was assessed as described by Chen et al. [16]. Briefly, each slide was randomly sampled in 5 locations, and CVF was defined as (blue stained area/total area) × 100. CVF for each patient was then recorded as the mean CVF of the 5 randomly sampled locations on the slide.

### Surgeon assessment of fibrosis

Qualitative assessment of tumor texture, included in operative notes per standard practice, was extracted for each patient. Fibrosis is reported in the narrative portion of the operative note and is rated on a scale from 0 to 3, where 0 = no fibrosis, 1 = mild fibrosis, 2 = moderate fibrosis, and 3 = extensive fibrosis. Reports are completed within 2 h of surgery and represent an accurate sense of surgical impression. Based on the information extracted from this description, each tumor was then assigned a consistency rating of 1–5 using a previously validated scale (Table 1) [25].

**Table 1:** Five-point scale for grading pituitary adenoma consistency

Grade	Description
1	Cystic or hemorrhagic tumor consistency
2	Soft tumor consistency; freely suckable tumor; minimal curettage required
3	Average tumor consistency: partially suckable tumor, requires some curettage or mechanical debulking; tumor readily descends from suprasellar space
4	Firm tumor consistency: not suckable, curettage or mechanical debulking required; tumor does not readily descend from suprasellar space; extracapsular technique typically required
5	Extremely firm or calcified tumor; not curettable, requires sharp or en bloc removal

Reprinted with permission from Rutkowski MJ, et al. *J Neurosurg*. 2020;134(6):1800–1807 [25]

### Assessment of surgical remission

Serum PRL values before and after surgery were compiled, with a PRL level above the upper limit of normal (ULN) in the hospital reference lab indicative of active disease. Normal PRL levels was 3.5–19.4 ng/mL for males and 5.0–26.5.0.5 ng/mL for females. Surgical remission was defined as PRL level at or below the ULN without use of DA. In patients lost to follow up after 3 months, early remission was defined as a PRL level < 3 ng/mL [26].

### Statistical analysis

Continuous variables were summarized using mean and standard deviation (SD) or median and range. Categorical variables were presented as frequencies and percentages. Associations between CVF and risk factors, including DA used, total duration of DA used, cumulative CAB dose, cumulative BRC dose, CS invasion, surgeon assessment of fibrosis, and tumor size were examined using Pearson’s correlation coefficients. One-way analysis of variance (ANOVA) test was used to compare mean CVF values across different categories of DA used and surgeon assessment of fibrosis, and Fisher’s exact test was used to test associations between categorical variables such as DA used and remission status. Student’s t-test or Mann–Whitney U test (two-sided) was used for continuous variables, depending on data distribution. Logistic regression analysis was performed to evaluate the association between remission status and the above-mentioned risk factors. Odds ratios (OR) with 95% confidence intervals (CIs) were estimated for both univariable and multivariable models, adjusting for potential confounders. Variables with a p value < 0.2 in the univariable analysis were included in the exploratory multivariable model. P values < 0.05 are considered statistically significant. All statistical analyses were conducted using R software version 4.3.3 [27].

## Results

### Patient characteristics

**Fig. 2:**
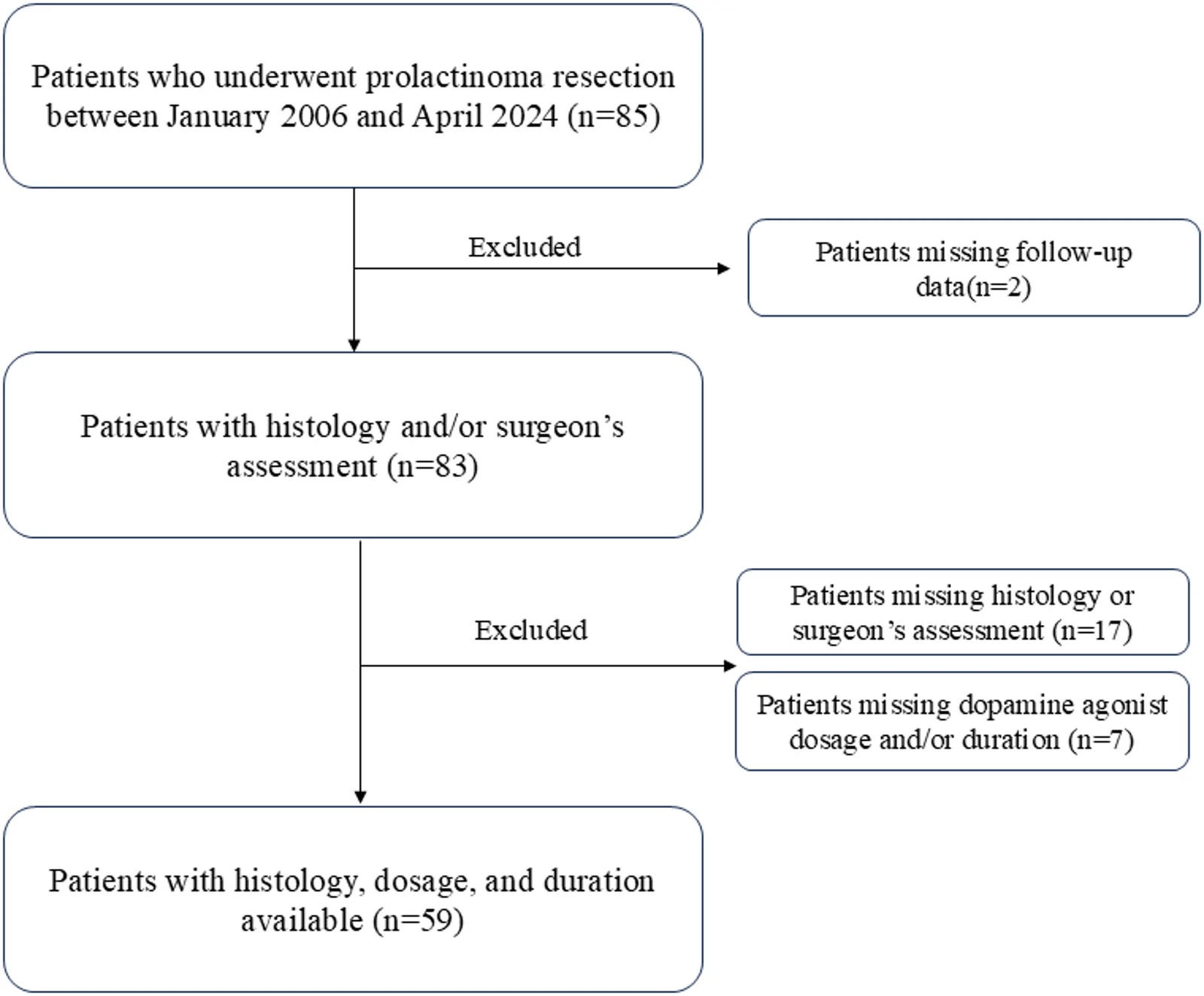
Subject inclusion and analysis flowchart

We identified 85 patients who underwent surgery for prolactinoma with subsequent follow-up assessment of remission during the study period (Fig. 2). Twenty-six patients were excluded because of missing histology and surgeon fibrosis assessment (*n* = 2), missing histology only (*n* = 17), or insufficient information to reliably determine exact dose or duration of DA used (*n* = 7). The final analysis cohort comprised 59 patients with complete data and follow up of at least 3 months, of whom 37 were pretreated with DA and 22 underwent surgery only. Last PRL level was recorded at least 1 year after surgery in 48 patients, and between 3 months and 1 year after surgery in 11 patients. Two patients were lost to follow up after 3 months.

Median postsurgical follow-up was 725 days (range, 90–5453) in the DA + group and 482 days (range, 90–5867) in the DA- group. Demographic features, presenting symptoms, and tumor size were generally similar between the two groups, except for more females in the DA + group (70.3% vs. 40.9%, *p* = 0.03) (Table 2).

**Table 2:** Patient characteristics

	DA+	DA-	*P* value
	(*n* = 37)	(*n* = 22)	
*Demographics*			
Female sex, n (%)	26 (70.3)	9 (40.9)	0.03
Age at surgery, median years [range]	32 [19–64]	38.5 [13–70]	0.8
Time from surgery to last follow up, median [range] days	725 [90–5453]	482 [90–5867]	0.17
*Clinical presentation*,* n (%)*			
Menstrual abnormalities, females	22 (84.6)	9 (100.0)	0.2
Headache	17 (45.9)	12 (54.5)	0.5
Galactorrhea, females	17 (65.4)	3 (33.3)	0.01
Vision loss/visual field deficit	9 (24.3)	7 (31.8)	0.5
Weight gain	9 (24.3)	7 (31.8)	0.4
Fatigue	5 (13.5)	6 (27.3)	0.3
Low libido	3 (8.1)	5 (22.7)	
Impaired memory	2 (5.4)	1 (4.5)	0.6
Erectile dysfunction, males	0 (0)	2 (15.4)	0.02
Other symptoms	14(64%)	8(22%)	0.001
*Imaging*,* n (%)*			
Macroadenoma	27 (73.0)	17 (77.3)	0.9
Microadenoma	9 (24.3)	4 (18.2)	0.8
Giant adenoma	1 (2.7)	1 (4.5)	0.6
Cavernous sinus invasion	15 (40.5)	13 (59.1)	0.4
*PRL levels*,* median ng/mL [range]*			
Pre-surgery PRL	83.9 [1.7–1965.0]	104.3 [11.5–7132.5.5.5]	0.1
Post-surgery PRL	3.6 [0.6–209.8.6.8]	6.9 [0.9–2551.8.9.8]	0.1
Most recent PRL	11.8 [0.6–102.9.6.9]	6.7 [0.6–104.9.6.9]	0.2
*Presurgical DA treatment*			
CAB only, n (%)	29 (78.8)	-	> 0.9
CAB + BRC, n (%)	8 (21.6)	-	
DA duration, median [range] days	570 [16–7830]	0 [0–21]	
*Cumulative DA dose*,* median [range]*			
CAB	79.32 [5.43–6711.43.43.43]	0 [0–2.14.14]	> 0.9
BRC	1265 [150–6875]	0[0–0]	> 0.9
*Indication for surgery*,* n (%)*			
Medication intolerance	17 (45.9)	1 (4.5)	
DA resistance	19 (51.4)	0 0)	
Patient preference	15 (40.5)	16 (72.7)	
Optic chiasm decompression	2 (5.4)	2 (9.1)	
Poor medication compliance	1 (2.7)	0 (0)	
Desired pregnancy, females	0 (0)	0 (0)	
Size of adenoma	0 (0)	3 (13.6)	
Age	0 (0)	2 (9.1)	
Amenable tumor	0 (0)	1 (4.5)	
Drain cyst	0 (0)	1 (4.5)	
Preserve normal gland function	0 (0)	1 (4.5)	
Sinus invasion	0 (0)	1 (4.5)	
Unknown	0 (0)	1 (4.5)	
Uncertain etiology	1 (2.7)	0 (0)	
*Fibrosis*			
Surgeon rating, n (%)	3.9		
1	2 (5.4)	4 (18.2)	
2	1 (2.7)	9 (40.9_	
3	7 (18.9)	3 (13.6)	
4	14 (37.8)	3 (13.6)	
5	12 (32.4)	2 (9.1)	
Mean rating	3.9	2.6	**0.002**
CVF	43.0 (0.3–84.5)	34.2 (4.5–47.9)	**0.045**

Values in bold are statistically significant. Mann-Whitney two-tailed U-test for non-parametric variables, one-tailed t-test for parametric values

*BRC* bromocriptine, *CAB* cabergoline, *CVF* collagen volume fraction, *DA* dopamine agonist, *PRL* prolactin. Surgeon rating as per Rutkowski MJ, et al. *J Neurosurg*. 2020;134(6):1800–1807 [20]

In the DA + group, 29 (78.8%) were treated with CAB only, and 8 (21.6%) received both CAB and BRC. Patients were treated for a median of 570 days (range, 16–7830). Median cumulative CAB dose was 79.3 mg in patients treated only with CAB; those who received CAB + BRC were treated with a median CAB dose of 1265 mg (range, 150–6875). The DA- group included 5 patients who were treated for less than 2 weeks, median cumulative DA dose in this group was 0 mg, but with a range of 0–2.1.1 mg.

Overall, 44 patients had macroadenomas (27 DA+, 17 DA-), 13 had microadenomas (9 DA+, 4 DA-), and 2 had giant adenomas (1 DA+, 1 DA-). CS invasion was noted in 15 (40.5%) patients in the DA + group and 13 (59.1%) patients in the DA- group. DA + patients exhibited lower PRL levels prior to surgery (median 83.9 vs. 104.3 ng/mL, respectively, NS) and lower PRL levels on postoperative day 1–2 (median 3.6 vs. 6.9 ng/mL, respectively, NS) compared with DA- patients. PRL levels at the last follow up were at or below the ULN in both groups (median 11.8 vs. 6.7 ng/mL, respectively).

Patient preference was the most common reason for surgery in the DA- group (72.7% DA- vs. 40.5 DA+), whereas intolerance to medication and DA resistance were most common in the DA + group (45.9% DA + vs. 4.5% DA- and 51.4% DA + vs. 9.1% DA-, respectively).

### Analysis of remission rates

Remission rates were similar between the groups (63.6% DA- vs. 45.9% DA+), regardless of type of DA used or tumor size (Table 3). Compared to DA + patients, remission rates were higher in DA- patients with microadenomas (100% vs. 77.8%), although the difference was not significant; rates were similar in those with macroadenomas (58.8% vs. 55.5%) and giant adenomas (0% vs. 0%). Analysis of patients who received only CAB (*n* = 29) vs. those who received BRC in addition to CAB (*n* = 8) also did not demonstrate any significant differences between groups.

**Table 3:** Remission rates

	Remission, *n* (%)	No remission, *n* (%)	*P* value
Total all groups	31 (52.5)	28 (47.5)	
*All*			0.5
DA- (*n* = 22)	14 (63.6)	8 (36.3)	
DA+ (*n* = 37)	17(45.9)	20(44.1)	
CAB only (*n* = 29)	13 (44.8)	16 (55.2)	
CAB + BRC (*n* = 8)	4 (50.0)	4 (50.0)	
*Microadenoma*			0.5
DA- (*n* = 4)	4 (100)	0 (0)	
DA+ (*n* = 9)	7(77.8)	2(22.2)	
CAB only (*n* = 8)	6 (75.0)	2 (25.0)	
CAB + BRC (*n* = 1)	1 (100)	0 (0)	
*Macroadenoma*			0.7
DA- (*n* = 17)	10 (58.8)	7 (41.2)	
DA+ (*n* = 27)	15(55.5)	12(44.5)	
CAB only (*n* = 20)	12 (60.0)	8 (40)	
CAB + BRC (*n* = 7)	3 (42.8)	4 (57.2)	
*Macroadenoma + Giant*			> 0.9
DA- (*n* = 18)	10 (55.5)	8 (44.5)	
DA+ (*n* = 28)	15(53.6)	13(46.4)	
CAB only (*n* = 21)	12 (57.1)	9 (42.9)	
CAB + BRC (*n* = 7)	3 (42.8)	4 (57.2)	

Fisher’s exact test for 2 × 2 and chi-square for other contingency tables

*BRC* bromocriptine, *CAB* cabergoline, *DA* dopamine agonist

### Analysis of tumor fibrosis

**Fig. 3:**
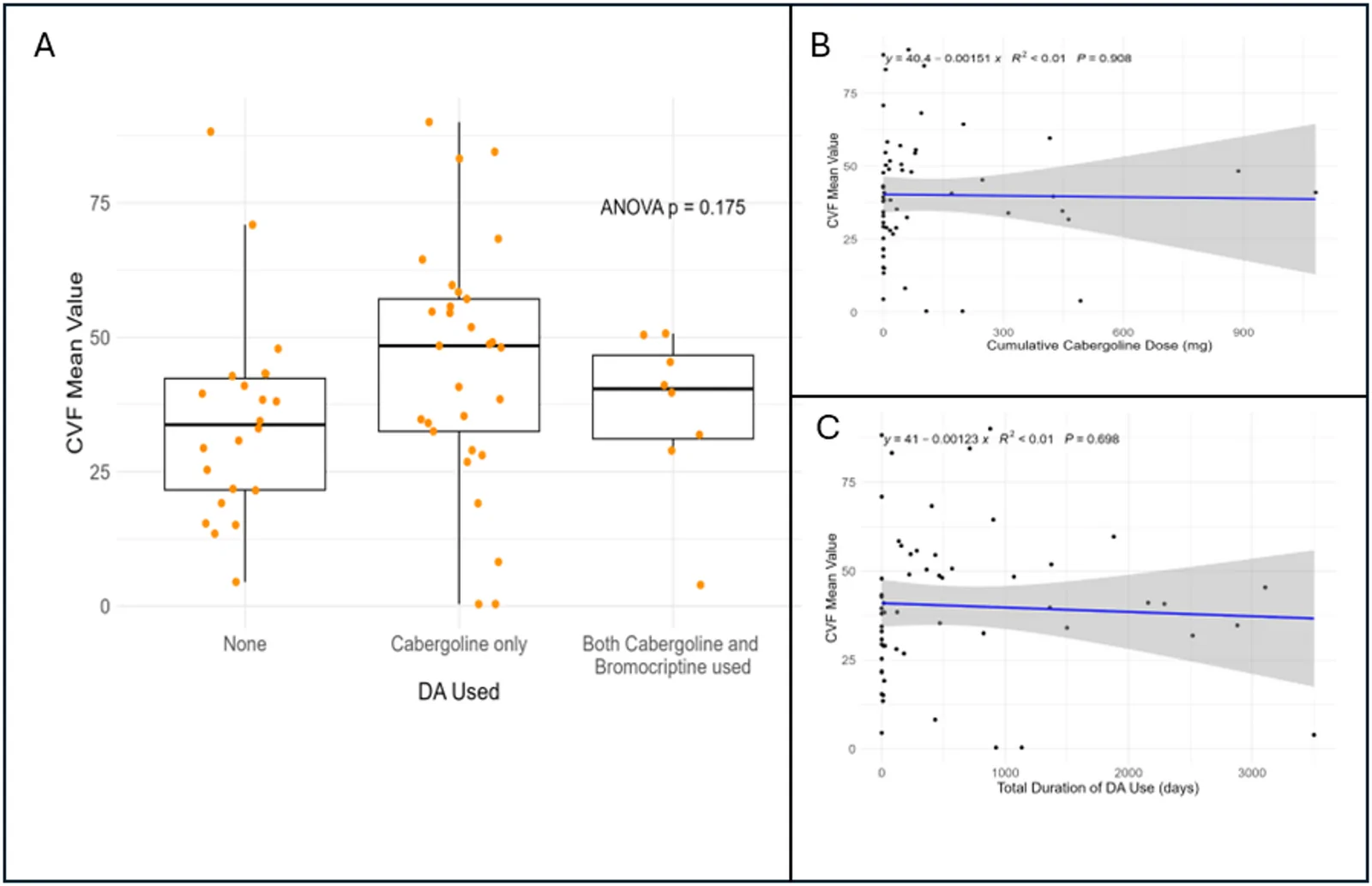
(**A**) Distribution of all CVF values across all tumors. There was wide distribution of CVF values when analyzed by type of DA used (none, CAB only, CAB + BRC). Tumors of CAB + patients had a mean higher CVF, but the difference was not significant compared to no pretreatment or pretreatment with CAB + BRC (*p* = 0.175, ANOVA). (**B**-**C**) Relationship between CVF and (**B**) cumulative CAB dose and (**C**) total duration of DA use. Neither cumulative dose nor duration correlated with CVF

Examination of CVF values showed some degree of fibrosis was present even in patients with no presurgical exposure to DA (Fig. 3A). Mean CVF was higher in the DA + group (43.0% [range:0.3–84.5]) than in the DA- group (34.2% [range:4.5–47.9], *p* = 0.045) (Table 2). Univariable analysis demonstrated no correlation between CVF and cumulative dose of CAB (*p* = 0.908) (Fig. 3B) or DA treatment duration (*p* = 0.698) (Fig. 3C) or between mean CVF and remission (*p* = 0.8) (Table 4).

**Table 4:** Predictors of remission

		Univariable analysis			Multivariable analysis		
Characteristic	*N*	OR	95% CI	*P* value	OR	95% CI	*P* value
DA total duration	59	1.00	1.00, 1.00	0.7			
CAB cumulative dose	59	1.00	1.00, 1.00	0.4			
CVF, mean	59	1.00	0.98, 1.03	0.8	1.01	0.97, 1.06	0.6
DA used	59						
None		—	—				
CAB only		2.15	0.70, 6.93	0.2			
CAB + BRC		1.75	0.33, 9.41	0.5			
Prolactinoma size	59						
Microadenoma		—	—		—	—	
Macroadenoma		6.60	1.54, 46.0	**0.022**	1.43	0.17, 13.8	0.7
Giant*		> 100	0.00, NA	> 0.9	> 100	0.00, NA	> 0.9
Cavernous sinus invasion	59						
No		—	—		—	—	
Yes		10.3	3.27, 36.8	**< 0.001**	6.42	1.12, 51.1	0.051
Sex	59						
Female		—	—		—	—	
Male		5.30	1.77, 17.4	**0.004**	0.97	0.18, 4.74	> 0.9
Age at surgery	59	1.03	0.99, 1.07	0.2			
Pre-operative PRL, ng/mL	57	1.00	1.00, 1.00	0.2			
Immediate post-op PRL, ng/mL	58	1.07	1.02, 1.13	**0.014**	1.05	1.01, 1.14	0.083
Surgeon assessment of fibrosis	59	1.40	0.94, 2.16	0.11	1.55	0.87, 3.02	0.2
Pre-treated with any DA	59						
No		—	—				
Yes		2.06	0.71, 6.29	0.2			

Values in bold are statistically significant

*CI* confidence interval, *CVF* collagen volume fraction, *DA* dopamine agonist, *PRL* prolactin, *OR* odds ratio

*Due to small number of cases (*n* = 2), not statistically valid measure of true OR

Surgeon assessment of fibrosis is shown in Table 2. The DA + group demonstrated significantly higher fibrosis ratings than did the DA- group (*p* = 0.002). These ratings were a significant predictor of CVF on univariable regression (*p* = 0.005), indicating that higher CVF scores were noted in patients with a higher surgeon-assessed degree of fibrosis overall. Yet, further analysis showed only a mild correlation between CVF and surgeon assessment of fibrosis (Pearson *r* = 0.21). One-way ANOVA indicated that for the overall group (DA + and DA-) and for the DA- group alone, CVF and surgeon assessment of fibrosis categories were correlated (*p* = 0.03 and *p* = 0.02, respectively), but CVF and surgeon assessment of fibrosis were not correlated in DA + patients alone (*p* = 0.31). Surgeon assessment of tumor fibrosis also did not predict remission based on univariable analysis (OR 1.4, *p* = 0.11). CVF was not significantly different in those with and without CS invasion (one-sided t-test, *p* = 0.12).

We also investigated other factors as possible predictors of remission (Table 4). On univariable analysis, CS invasion (*p* < 0.001) and tumor size (*p* = 0.022) predicted remission. However, on multivariable logistic regression analysis, no individual factor predicted remission status at last follow up. Surgeon assessment of fibrosis, CVF, and use of DA did not predict remission.

## Discussion

This study represents the first rigorous effort to correlate the dose and duration of presurgical CAB therapy with both the extent of prolactinoma fibrosis and surgical outcomes.

We considered the degree of fibrosis as qualitatively assessed intraoperatively by the surgeon during the operation and as quantitatively measured based on collagen fraction from MTC staining to minimize the inherent biases with surgeon-based rating scales. We conclude that neither presurgical DA use, surgeon assessment of fibrosis, nor CVF assessment of fibrosis independently predicted remission.

The effect of presurgical DA use on tumor fibrosis and surgical outcomes in patients with prolactinoma has mostly focused on treatment with BRC [1, 12, 16]. True quantitative data on presurgical CAB use have been relatively limited [14, 17]. Prior reports often lacked detailed information regarding treatment dose, duration, or the specific DA utilized [28], thereby precluding meaningful correlations between CAB exposure and fibrosis. Some evidence suggests that CAB may increase the number of stromal cells within prolactinomas, a potential histological correlate of fibrosis [15], but this effect does not appear to be dose dependent.

We aimed to address these knowledge gaps by examining a large group of prolactinomas that were pretreated with only CAB or with a combination of CAB and BRC. Our results indicate no clear correlation between CVF and either DA dose or treatment duration.

Despite a significant difference in tumor fibrosis identified between the DA + and DA- groups based on subjective surgeon assessment ratings of tumor consistency, we found that CVF, a quantitative measure of fibrosis, demonstrated no clear correlation with DA use. This discrepancy highlights the fact that surgeon assessment of fibrosis is likely influenced by observational bias at the time of the procedure and may lead to over- or under-estimation of tumor consistency based on perceived extent of surgical resection. CVF likely provides a far more objective histological measure, and its routine use in studies evaluating the relationship between tumor consistency and surgical outcome seems justified.

Consistent with previous reports [1, 2, 11, 24], we show that tumor size predicted remission rates, as patients with microadenomas had a higher rate of long-term remission than did those with macroadenomas or giant tumors. We also observed that a higher proportion of patients with microadenoma who were not pretreated with CAB achieved remission compared with those who received pretreatment, although the difference did not reach statistical significance. It is possible that this trend might become significant in a larger cohort of microadenomas, as suggested by findings from a meta-analysis [29].

Among patients with macroadenomas, remission rates did not differ based on either the dose or duration of DA therapy. Only CS invasion predicted surgical cure, which is consistent with a previous report [26]. It is unlikely that we included patients with early remission but later recurrence, as median duration of follow up was > 2 years post-surgery and mean PRL was normal without DA use at last follow up. Our results thus provide a good estimate of long-term outcomes.

DA resistance, manifested by an increase in PRL levels despite increasing DA doses, is a common reason for surgical intervention [30]. For intrasellar microadenomas, our data support the view that CAB can be used as a first-line therapy, without the fear that prolonged use will markedly reduce the chances for surgical cure at a later point should DA resistance or intolerance occur. Thus, a decision to operate should not depend on whether a patient has been pretreated with CAB, regardless of the duration or cumulative dose.

Our observation that a subset of patients who had never received CAB nonetheless exhibited elevated tumor CVF and fibrosis indicates that these features cannot be solely attributed to the use of DA. Instead, these findings support the concept that development of pituitary tumor fibrosis is multifactorial and that our understanding of its drivers is incomplete. Recent work has highlighted the importance of interactions between pituitary tumor cells and the surrounding microenvironment, particularly immune cells and cancer-associated fibroblasts (CAFs) [31]. CAFs secrete inflammatory cytokines such as interleukin (IL)−2, IL-6, and IL-8 as well as growth factors including vascular endothelial growth factor (VEGF)-A and fibroblast growth factor (FGF)−2, all of which promote fibrosis via inflammatory or remodeling pathways. In addition, tumor associated macrophages release transforming growth factor (TGF)-ꞵ, a multifactorial cytokine that regulates apoptosis, inflammation, and extracellular matrix deposition and fibrosis [32]. TGF-β1 mediates the inhibitory effect of dopamine in normal lactotroph cells [33]. TGF-ꞵ1 expression and mRNA levels in prolactinomas has been correlated with pituitary tumor fibrosis and collage deposition [34], and TGF-β1 is markedly downregulated in DA-resistant prolactinomas. Together, these data support an independent role for TGF-β1 in development of lactotroph tumor fibrosis [35].

Furthermore, CAB has been shown to modulate immune pathways. A recent study demonstrated increased infiltration of CD4 + and CD8 + T cells as well as CAF activation in prolactinomas among patients previously treated with CAB versus those not treated with CAB [15].These observations suggest that prolactinoma fibrosis likely reflects converging actions of tumor-intrinsic signals, stromal and immune-cell interactions, and, at least in some patients, additional DA-enhancing effects.

The high surgical cure rates, low morbidities, and potential cost-efficacy observed for intrasellar microadenomas justify upfront or early surgery as a viable option, a view that has been heavily emphasized in recent years [10, 36]. However, our data do not support the view that upfront surgery should be performed to avoid potential effects of tumor fibrosis related to a course of CAB therapy. Rather, our data indicate that an individualized approach to treatment selection can be implemented without concern for losing the option for the alternate therapy at a later date, a view also supported by recent publications [37].

For macroadenomas, as the cure rate or degree of fibrosis does not appear to be dependent on duration of DA use, CAB may be used to reduce tumor volume and normalize PRL levels before surgery. In these patients, even if postsurgical remission is not achieved, some studies suggest lower doses of DA may be needed to control PRL levels after surgical debulking [10, 36, 38–40].

We confirmed that CS invasion remains the most important predictor of surgical remission. Given the high rates of DA responsiveness for most prolactinomas and the potential for increased morbidity with surgery in the CS, surgery is generally not recommended in these patients [2]. Furthermore, CS invasion did not correlate with degree of tumor fibrosis, indicating that presurgical DA use would not preclude patients from considering surgery in this setting. As more surgeons become comfortable with techniques that enable safer medial cavernous wall resections [40], it is possible that pretreatment with DA and subsequent surgery could prove beneficial in patients with CS invasion. Further data will be needed to validate this approach.

We recognize limitations in our study. Although our overall cohort is relatively large for surgically removed prolactinomas, subset analyses were performed on smaller groups, which might have affected our ability to detect small differences. Determination of the exact doses of DA taken by patients over many years is also a challenge, as it is not always clear how compliant patients were and whether there were dose escalations or other changes. Nonetheless, we were as meticulous as possible in confirming the data, and the likelihood of variability in DA use affecting the dataset is low. Finally, we included some patients with both CAB and BRC use. While no differences were noted, the small numbers in this group may preclude our ability to detect differences between combined use with CAB and BRC vs. those receiving CAB alone.

This study is the largest and most comprehensive evaluation of CAB-associated fibrosis and its impact of on surgical outcomes in patients with prolactinoma. We found that pretreatment with CAB has no significant impact on overall surgical outcomes for prolactinoma patients, and that the dose and duration of treatment are not predictive of the degree of fibrosis encountered at surgery. Thus, our results suggest that patients treated with CAB should not be precluded from consideration for subsequent surgery. We also note that surgeon assessment of fibrosis was not highly correlated with quantitative measures such as CVF and should likely be interpreted with caution.

As surgical intervention for prolactinoma appears to be gaining in popularity with the advent of safer endoscopic techniques, our results offer important guidance to clarify selection of appropriate treatment options for patients with prolactinoma.

## Data Availability

Restrictions apply to the availability of some data analyzed during this study to preserve patient confidentiality. The corresponding author will on request detail the restrictions and any conditions under which access to these data may be provided.
