# Correction: Long-term cabergoline use does not predict degree of prolactinoma fibrosis nor significantly impact surgical outcomes

**DOI:** 10.1007/s11102-026-01734-1

**Published:** 2026-08-23

**Authors:** Adam N. Mamelak, Rachel Fox, Yaakov Rosenberg, Daniel Gomez, Yujie Cui, Anat Ben Shlomo, Artak Labadzhyan, Ning-Ai Liu, Vivien Bonert, Odelia Cooper

**Affiliations:** 1 Department of Neurosurgery, Cedars-Sinai Medical Center, 127 S. San Vicente Blvd., Ste. A6313, Los Angeles, CA 90035 USA; 2 Pituitary Center, Department of Medicine, Cedars-Sinai Medical Center, Los Angeles, CA USA; 3 Biostatistics Shared Resource, Cedars-Sinai Medical Center, Los Angeles, CA USA

**Correction: Pituitary (2026) 29:27**

**https://doi.org/10.1007/s11102-025-01628-8**

In this article the author’s name Anat Ben-Shlomo was incorrectly written as Anat Ben Shlomo.

The original article has been corrected.

